# The Polyomavirus Episteme: A Database for Researchers

**DOI:** 10.1128/MRA.00108-21

**Published:** 2021-04-08

**Authors:** María José Andrade, Gau Shoua Vue, Michael J. Imperiale

**Affiliations:** a Swarthmore College, Swarthmore, Pennsylvania, USA; b Department of Microbiology and Immunology, University of Michigan, Ann Arbor, Michigan, USA; Indiana University, Bloomington

## Abstract

*Polyomaviridae* members are DNA viruses that infect a variety of species. Since the first polyomavirus was isolated in 1953, technological advancements have led to the discovery of many polyomaviruses in multiple species. The Polyomavirus Episteme curates data about each polyomavirus, drawing from public databases to present known taxonomic, genomic, and clinical information about polyomaviruses.

## ANNOUNCEMENT

The first polyomavirus was isolated in 1953 from tumors in laboratory mice, and the name of the virus was thus composed from the Greek roots poly-, meaning many, and -oma, meaning tumors (1, 2). Polyomaviruses are host specific and have been found in mammals, birds, and some fish, causing symptomatic infections or cancer in some hosts (3). Importantly for humans, they have been known to cause illness in immunocompromised patients. Viruses belonging to the family *Polyomaviridae* have nonenveloped, double-stranded circular DNA genomes that are associated with histones taken from their host (4). The viruses can express five to seven proteins, including three viral capsid proteins (VP1, VP2, and VP3), as well as two proteins involved in cell cycle regulation and replication of DNA, called the large tumor antigen (LT) and small tumor antigen (ST) proteins (3). The transcription of these proteins is temporally regulated; early proteins are involved in replication and are expressed prior to the onset of DNA replication, and late proteins are involved in capsid formation and are expressed after DNA replication has begun.

Polyomaviruses are very diverse. A complex mixture of evolutionary processes related to polyomavirus host specificity and recombination might have led to this diversity (5). In recent years, many novel polyomaviruses have been discovered due to advancements in cloning and sequencing methods (6). These advancements have created a need for an integrated resource with information on all polyomaviruses. An episteme, a Greek term referring to a system of scientific knowledge, captures the purpose of such a project. Following the model of a similar resource created for viruses belonging to the *Papillomaviridae* family (7), the Polyomavirus Episteme (https://sites.google.com/umich.edu/polyomavirusepisteme) has been created as a resource for polyomavirus sequence data and analysis, with curated information on all discovered polyomaviruses.

**FIG 1:**
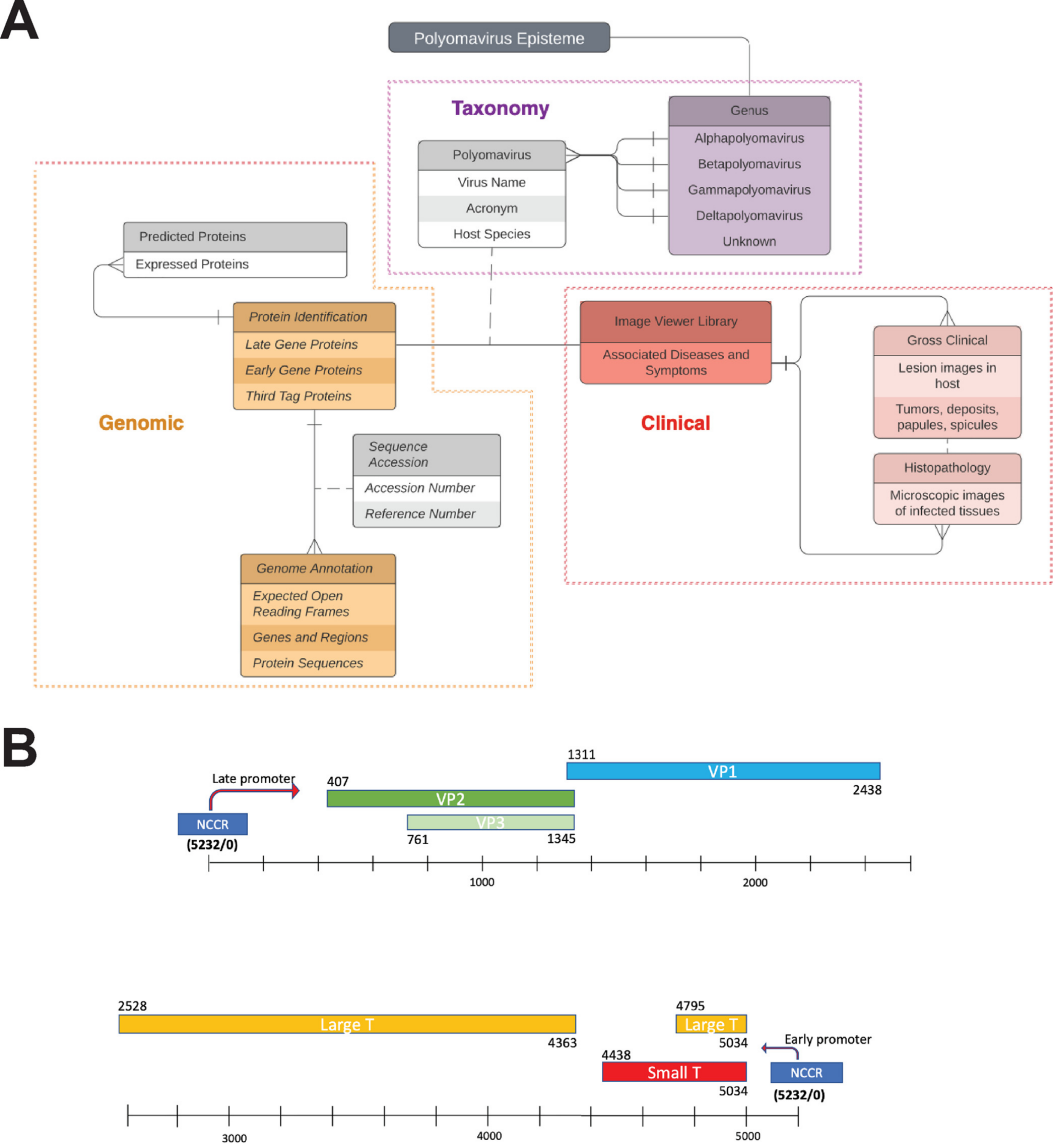
(A) Structure of the database, presented as an entity-relationship diagram. (B) Example of an annotated late coding region, from trichodysplasia spinulosa-associated polyomavirus.

The Polyomavirus Episteme includes taxonomic, genomic, and clinical information about each polyomavirus (Fig. 1A). The data were collected from public sources, including PubMed and the NCBI nucleic acid and protein databases.

Taxonomic data include information about the genus, species, and natural host of each virus. The genus of a polyomavirus is determined from phylogenetic relationships of LT proteins (8). There are four genera, each with a restricted natural host range, and there are more than 100 species. Some viruses have not yet been assigned to a genus and are classified under “unknown genus.”

Genomic data include data on the early and late proteins expressed by a specific reference strain of each virus. Polyomavirus genomes typically encode two regulatory proteins during early infection, the LT and ST proteins, which are called the early proteins. The three capsid proteins, VP1, VP2, and VP3, are expressed after the onset of viral DNA replication and are thus called late proteins. VP1 enables attachment and entry of virus into host cells, and VP2 and VP3, which have overlapping open reading frames (ORFs) (with VP3 being identical to the C terminus of VP2), have nuclear localization signals that, once virion disassembly is initiated in the cytosol, guide the particles into the nucleus, where further disassembly occurs (3, 9). Most polyomaviruses have these ORFs, but many polyomaviruses express additional early and late proteins. The genomes of most polyomaviruses are approximately 5,000 bp and have a noncoding control region (NCCR) located between the early and late regions, which contains the origin of DNA replication and the early and late promoters (9).

Through analysis of the ORFs of each virus, which have been curated from public databases such as the NCBI databases and from relevant publications, each viral genome has been annotated to show the late and early regions. Encoded proteins and some important motifs are annotated with their genome locations by base pair. The gaps in the annotation of LT proteins and some other proteins in certain polyomaviruses indicate spliced mRNA. The NCCR has high sequence variability among polyomaviruses, and it is not included in the annotation of all viruses. An example of annotation is shown in Fig. 1B.

Polyomaviruses can cause symptomatic infections and tumors in human and veterinary hosts. The Polyomavirus Episteme includes a library of gross clinical and histopathology images of lesions and diseases associated with each polyomavirus. The relevant publications are specified under each image.

The Polyomavirus Episteme uses Google Sites, with underlying code written in HTML. The annotations were manually edited on the basis of the NCBI databases and were confirmed using additional publications in the scientific literature to ensure that all expected ORFs were included.

The Polyomavirus Episteme has been curated to aid the study of polyomaviruses by integrating and linking multiple resources and facilitating navigation through the growing number of discoveries of novel virus types. As interests in polyomavirus biology and nonhuman infections grow, the Polyomavirus Episteme can become a resource for facilitating the annotation and classification of novel viral genomes. We hope to periodically update and expand the database annotation of genomes to include transcriptional promoters, enhancers, and binding sites for key *trans*-acting factors. We also hope to receive feedback from people in the field who may have recommendations for useful features that could be incorporated in the future.

### Data availability.

The episteme can be found at https://sites.google.com/umich.edu/polyomavirusepisteme.
